# Systemic anticancer therapy for urothelial carcinoma: UK oncologists’ perspective

**DOI:** 10.1038/s41416-023-02543-0

**Published:** 2024-01-08

**Authors:** Robert J. Jones, Simon J. Crabb, Mark Linch, Alison J. Birtle, John McGrane, Deborah Enting, Robert Stevenson, Kin Liu, Bihani Kularatne, Syed A. Hussain

**Affiliations:** 1 University of Glasgow, Beatson West of Scotland Cancer Centre, Glasgow, UK; 2 School of Cancer Sciences, University of Southampton, Southampton, UK; 3 UCL Cancer Institute, University College London, London, UK; 4 Rosemere Cancer Centre, Lancashire Teaching Hospitals NHS Foundation Trust, Preston, UK; 5 University of Central Lancashire, Lancaster, UK; 6 University of Manchester, Manchester, UK; 7 Royal Cornwall Hospital NHS Trust, Truro, UK; 8 Guy’s and St Thomas’ NHS Foundation Trust, London, UK; 9 University Hospital Birmingham, Birmingham, UK; 10 Merck Serono Ltd., an affiliate of Merck KGaA, Feltham, UK; 11 Pfizer Ltd., Tadworth, UK; 12 University of Sheffield and Sheffield Teaching Hospitals NHS Foundation Trust, Sheffield, UK

**Keywords:** Bladder cancer, Targeted therapies

## Abstract

Urothelial carcinoma (UC) is a common cancer associated with a poor prognosis in patients with advanced disease. Platinum-based chemotherapy has remained the cornerstone of systemic anticancer treatment for many years, and recent developments in the treatment landscape have improved outcomes. In this review, we provide an overview of systemic treatment for UC, including clinical data supporting the current standard of care at each point in the treatment pathway and author interpretations from a UK perspective. Neoadjuvant cisplatin-based chemotherapy is recommended for eligible patients with muscle-invasive bladder cancer and is preferable to adjuvant treatment. For first-line treatment of advanced UC, platinum-eligible patients should receive cisplatin- or carboplatin-based chemotherapy, followed by avelumab maintenance in those without disease progression. Among patients unable to receive platinum-based chemotherapy, immune checkpoint inhibitor (ICI) treatment is an option for those with programmed death ligand 1 (PD-L1)–positive tumours. Second-line or later treatment options depend on prior treatment, and enfortumab vedotin is preferred after prior ICI and chemotherapy, although availability varies between countries. Additional options include rechallenge with platinum-based chemotherapy, an ICI, or non–platinum-based chemotherapy. Areas of uncertainty include the optimal number of first-line chemotherapy cycles for advanced UC and the value of PD-L1 testing for UC.

## Introduction

Bladder cancer is the 11th most common cancer in the UK and can be broadly categorised into three key stages: non–muscle-invasive, muscle-invasive, and locally advanced or metastatic disease [1, 2]. Each year, ≈10,300 new cases of bladder cancer are diagnosed, and ≈5600 deaths are attributed to bladder cancer, representing 3% of all UK cancer deaths [1]. Although most patients are diagnosed with localised disease (Stage I or II in ≈55% of patients in England) [3], ≈50% of patients who undergo radical treatment for muscle-invasive disease experience relapse and are likely to develop distant metastases [4, 5]. In addition, ≈10% of patients with bladder cancer in England have unresectable metastatic disease (Stage IV) at diagnosis [3]. The 5-year overall survival (OS) rate in patients across all disease stages is 53.8%, with survival rates decreasing with advancing stage [6]. One-year OS rates are 92.5% for Stage I, 73.6% for Stage II, 63.7% for Stage III, and 29.1% for Stage IV [6]. Risk factors for bladder cancer include smoking, older age, male sex, occupational exposure to aromatic amines or polycyclic aromatic hydrocarbons, and exposure to ionising radiation [1, 7]. Urothelial carcinoma (UC) accounts for 90% of bladder cancers, with the remaining 10% having a non-UC histology (i.e., squamous, small cell, sarcoma, or adenocarcinoma) [4]. In addition, although most cases of UC originate in the bladder, UC can also arise in the cells lining the urothelial tract in other sites, including the renal pelvis, ureter, and urethra [4]. Upper tract UC (UTUC; originating in the renal pelvis and ureter) accounts for ≈5–10% of all UC cases and ≈20–30% of metastatic UC cases [4, 8–12].

Perioperative systemic anticancer drug treatment is recommended for patients with muscle-invasive bladder cancer who are undergoing radical cystectomy or radiotherapy with curative intent. In addition, systemic anticancer treatment is standard of care for patients diagnosed with unresectable locally advanced or metastatic UC (termed advanced UC hereafter), either de novo or following relapse after treatment for earlier-stage disease [4, 8, 13], with the aims of extending survival and improving symptom control. Options for systemic anticancer treatment in UC have increased in recent years with the advent of immune checkpoint inhibitors (ICIs), antibody-drug conjugates (ADCs), and fibroblast growth factor receptor (FGFR) inhibitors, which have been approved for different patient populations in various countries worldwide. However, specific treatment options in individual countries vary depending on approval and reimbursement. In the UK, new treatments to be administered within National Health Service care must be included as part of clinical guidance published by the National Institute for Health and Care Excellence (NICE) for England, which is also taken into account for Wales and Northern Ireland (subject to advice from the All Wales Medicines Strategy Group and Department of Health, respectively), or accepted for use by the Scottish Medicines Consortium (SMC) for Scotland. Thus, treatment decisions must consider clinical evidence and local approvals and guidance.

In this review, we provide an overview of systemic anticancer treatments that are approved by international guidelines and/or recommended by NICE or accepted for use by the SMC for patients with UC at different points in the treatment pathway. This is accompanied by summaries of relevant clinical data and author perspectives with a UK focus. We also discuss areas of uncertainty and highlight recent data and ongoing trials in UC that have the potential to change the treatment landscape.

## Neoadjuvant treatment of muscle-invasive bladder cancer

**Fig. 1:**
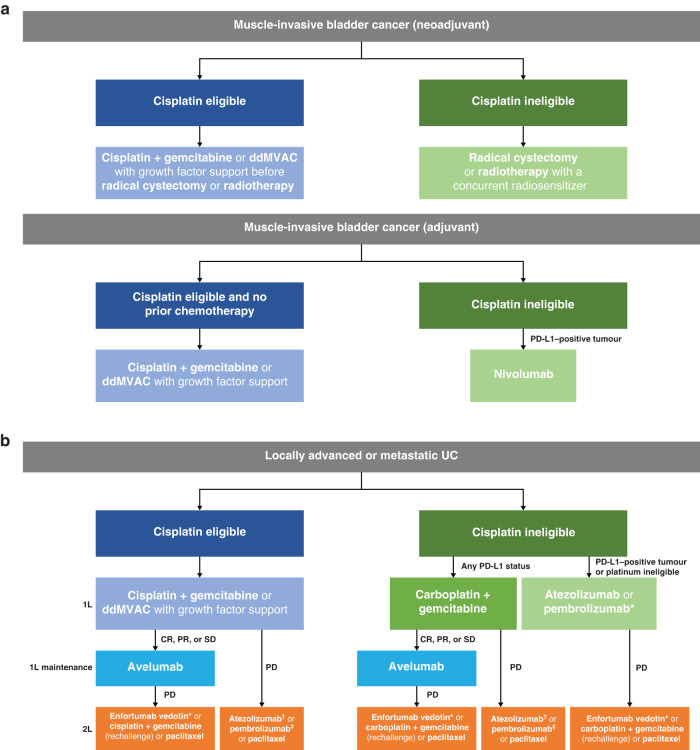
Systemic anticancer treatment options in the UK [8, 13, 14, 30, 31, 47, 50, 51, 59, 60, 63–65, 69, 70]. Treatment options for patients with (**a**) muscle-invasive bladder cancer and (**b**) locally advanced or metastatic UC based on NICE and SMC guidelines and author interpretation. 1L first line, 2L second line, CR complete response, ddMVAC dose-dense methotrexate, vinblastine, doxorubicin, and cisplatin, NICE National Institute for Health and Care Excellence, PD progressive disease, PD-L1 programmed death ligand 1, PR partial response, SD stable disease, SMC Scottish Medicines Consortium, UC urothelial carcinoma. *Not recommended by NICE or the SMC but approved by the European Medicines Agency. ^†^Not accepted for use by the SMC. ^‡^Accepted for use by the SMC.

In international guidelines, neoadjuvant cisplatin-based chemotherapy before radical cystectomy or radiotherapy is recommended for cisplatin-eligible patients with newly diagnosed muscle-invasive bladder cancer [4, 8]; it is also recommended by NICE (Fig. 1) [14]. This is supported by two randomised Phase 3 trials showing that neoadjuvant chemotherapy was associated with a reduced risk of death vs cystectomy alone (and/or radiotherapy in one of the trials) [15, 16]. Subsequently, a meta-analysis of 11 randomised trials of neoadjuvant platinum-based chemotherapy in patients with muscle-invasive bladder cancer found a significant OS benefit (equivalent to a 5% absolute improvement at 5 years) when platinum-based chemotherapy was added to local treatment (hazard ratio [HR], 0.86 [95% CI, 0.77–0.95]; *P* = 0.003); disease-free survival (DFS) was also significantly improved (HR, 0.78 [95% CI, 0.71–0.86]; *P* < 0.0001) [17]. There is no consensus on the optimal regimen. In the UK, 3-weekly cisplatin + gemcitabine is commonly used and is the control arm regimen in most ongoing international randomised trials. A recent randomised Phase 3 trial investigating perioperative dose-dense methotrexate, vinblastine, doxorubicin, and cisplatin (ddMVAC) vs cisplatin + gemcitabine did not meet its primary endpoint of progression-free survival (PFS) rate at 3 years; however, numerical improvements in 3-year PFS rate (64% vs 56%; HR, 0.77 [95% CI, 0.57–1.02]; *P* = 0.066) and 5-year OS rate (64% vs 56%; HR, 0.77 [95% CI, 0.58–1.03]; *P* = 0.078) were observed with ddMVAC, and disease-specific survival was significantly improved (HR, 0.63 [95% CI, 0.46–0.86]; *P* = 0.004) [18, 19]. Neoadjuvant chemotherapy is not recommended for cisplatin-ineligible patients, who generally proceed directly to radical treatment; an unmet need remains to improve outcomes in these patients [4]. Additionally, the role of neoadjuvant chemotherapy for patients with UTUC remains unclear due to a lack of data in this population.

Radiotherapy with a concurrent radiosensitizer is recommended as radical treatment for patients who are unable to undergo radical cystectomy, or as an alternative to cystectomy where bladder preservation is a goal (Fig. 1) [4]. Common radiosensitising options used in the UK include 5-fluorouracil + mitomycin or weekly gemcitabine, or carbogen (98% O_2_ and 2% CO_2_) + nicotinamide. In a randomised Phase 3 trial conducted in the UK, concomitant chemoradiotherapy with 5-fluorouracil + mitomycin improved locoregional control (HR, 0.61 [95% CI, 0.43–0.86]; *P* = 0.005) and invasive locoregional control (HR, 0.55 [95% CI, 0.36–0.84]; *P* = 0.006) vs radiotherapy alone, with a nonsignificant effect on DFS (HR, 0.78 [95% CI, 0.60–1.02]; *P* = 0.069) and OS (HR, 0.88 [95% CI, 0.69–1.13]; *P* = 0.3) [20, 21]. A UK randomised Phase 3 trial of carbogen + nicotinamide given with radical radiotherapy vs radiotherapy alone showed a non-statistically significant improvement in cystoscopic control (81% vs 76% at 6 months; *P* = 0.3 [primary endpoint]) but a significant improvement in OS (3-year OS, 59% vs 46%; *P* = 0.04) [22]. Real-world data from a retrospective study including three UK institutions were aligned with the results of this trial [23]. A randomised trial of cisplatin + radiotherapy vs radiotherapy alone as preoperative or definitive therapy showed improved local control rates (HR, 0.50 [90% CI, 0.29–0.86]; *P* = 0.036) [24], but this is less widely used in the UK. A single-arm Phase 2 trial in the UK showed that weekly gemcitabine and hypofractionated radiotherapy resulted in high rates of local control and response [25]; this regimen is used in some UK centres.

## Adjuvant treatment of muscle-invasive disease after radical cystectomy or nephroureterectomy

Neoadjuvant chemotherapy is the preferred approach in patients with bladder cancer. However, for patients who only become suitable for cisplatin after cystectomy, those who are upstaged from non–muscle-invasive to muscle-invasive disease at cystectomy, or those undergoing nephroureterectomy for muscle-invasive UTUC, adjuvant chemotherapy should be considered [8] and is recommended by NICE (Fig. 1) [14]. A randomised Phase 3 trial comparing immediate adjuvant cisplatin-based chemotherapy vs deferred adjuvant chemotherapy at relapse after cystectomy (EORTC 30994) was closed early due to poor accrual; therefore, it was under-powered to demonstrate an improvement in OS (primary endpoint; HR, 0.78 [95% CI, 0.56–1.08]; *P* = 0.13) [26]. Nonetheless, the trial showed improved PFS with immediate vs deferred adjuvant chemotherapy (HR, 0.54 [95% CI, 0.40–0.73]; *P* < 0.0001) [26]. A meta-analysis of data from this and other randomised trials comparing adjuvant cisplatin-based chemotherapy + local treatment vs local treatment alone showed that cisplatin-based chemotherapy improves OS (HR, 0.82 [95% CI, 0.70–0.96]; *P* = 0.02) and recurrence-free survival (HR, 0.71 [95% CI, 0.60–0.83]; *P* < 0.001) after cystectomy [27]. In UTUC, a randomised Phase 3 trial comparing adjuvant platinum-based chemotherapy vs surveillance after nephroureterectomy (POUT) found that DFS was significantly improved with platinum-based chemotherapy (HR, 0.48 [95% CI, 0.33–0.71]; *P* = 0.0003) [28, 29]. In this trial, patients with impaired renal function following nephroureterectomy could receive carboplatin instead of cisplatin [28]. Although not powered to detect a difference in OS, a positive trend was observed [29].

Nivolumab, an anti–programmed death 1 (PD-1) ICI, is an option as adjuvant therapy for patients with high-risk muscle-invasive UC who have received prior neoadjuvant cisplatin-based chemotherapy or who are unsuitable for adjuvant cisplatin (Fig. 1). It is recommended by NICE and accepted for use by the SMC for patients with programmed death ligand 1 (PD-L1)–positive tumours (≥ 1% expression on tumour cells; Dako 28-8 assay; Table 1), but it is not definitively recommended in European Society for Medical Oncology (ESMO) guidelines because OS data are not yet available [8, 30, 31]. Nivolumab adjuvant therapy (up to 12 months) was assessed in a randomised, double-blind, Phase 3 trial (CheckMate 274) in patients with high-risk muscle-invasive UC who had undergone radical surgery, including >40% who had received prior neoadjuvant cisplatin-based chemotherapy [32]. The primary endpoint was DFS, assessed in both the intention-to-treat population and patients with PD-L1–positive tumours. DFS was significantly longer with adjuvant nivolumab vs placebo, both in the intention-to-treat (HR, 0.70 [98.22% CI, 0.55–0.90]; *P* < 0.001) and PD-L1–positive populations (HR, 0.55 [98.72% CI, 0.35–0.85]; *P* < 0.001). Distant metastasis-free survival, a secondary endpoint, was also improved with nivolumab vs placebo in the intention-to-treat population (HR, 0.72 [95% CI, 0.59–0.89]) [32]. Although the trial included a subgroup of patients with UTUC, the trial was not powered to formally assess differences between arms in this subgroup [32], and subsequent changes in practice (use of carboplatin in cisplatin-ineligible patients) make it difficult to interpret the relevance of these data for patients with UTUC. Although CheckMate 274 met its primary endpoint in both the intention-to-treat and PD-L1–positive populations, marketing authorisation in Europe has only been granted for the PD-L1–positive population [30, 31]. Results from CheckMate 274 are in contrast to those of a randomised Phase 3 trial that compared adjuvant atezolizumab (anti–PD-L1) vs observation in a similar population (IMvigor010), which reported no significant improvement in DFS (primary endpoint; HR, 0.89 [95% CI, 0.74–1.08]; *P* = 0.24) or OS (HR, 0.85 [95% CI, 0.66–1.09]) [33]. Exploratory analyses, however, suggested that patients with detectable circulating tumour DNA following cystectomy had improved DFS and OS with atezolizumab vs observation, whereas no difference was seen in patients without [34]. An ongoing randomised Phase 3 trial (IMvigor011) is prospectively investigating atezolizumab vs placebo in patients with high-risk muscle-invasive UC who are subsequently found to have detectable circulating tumour DNA during follow-up post cystectomy.

**Table 1:** PD-L1 assays required for treatment access in the UK [31, 32, 47–49, 78].

ICI	Therapy type/patient population	Antibody	Assay	Cutoff
Nivolumab	Adjuvant therapy after neoadjuvant cisplatin-based chemotherapy or unsuitable for adjuvant cisplatin	Dako 28-8	TPS: PD-L1–positive TC/total TC	≥1%
Pembrolizumab*	1L treatment in those unsuitable or unwilling to receive platinum-based chemotherapy	Dako 22C3	CPS: PD-L1–positive TC & IC/total cells	≥10%
Atezolizumab^†^	1L treatment in those unsuitable or unwilling to receive platinum-based chemotherapy	Ventana SP142	Ventana IC algorithm: PD-L1–positive IC/total IC	≥5%

*1L* first line, *CPS* combined positive score, *IC* immune cell, *NICE* National Institute for Health and Care Excellence, *PD-L1* programmed death ligand 1, *SMC* Scottish Medicines Consortium, *TC* tumour cell, *TPS* tumour proportion score.

*Not recommended by NICE or accepted for use by the SMC [50, 51].

^†^Not accepted for use by the SMC.

## First-line treatment of de novo advanced UC

Potential options for first-line (1L) treatment of patients with advanced UC depend on whether patients are eligible for cisplatin-based chemotherapy, unsuitable for cisplatin but eligible for carboplatin-based chemotherapy, or unsuitable for any platinum-based chemotherapy (≈10% of patients) [35]. Definitions for cisplatin and platinum eligibility (discussed below) are based on consensus expert opinion [35–37]. In clinical practice, definitions provide guidance to consider eligibility, but other factors may influence treatment choice. In cisplatin-ineligible patients, PD-L1 status may also be relevant.

For patients eligible for cisplatin, defined as those with a good performance status (PS; e.g., Eastern Cooperative Oncology Group [ECOG] PS of 0–1) and adequate renal function (glomerular filtration rate [GFR] of 50–60 mL/min), cisplatin-based chemotherapy is the established 1L standard of care [8, 13, 37] and is recommended by NICE (Fig. 1) [14]. The most commonly used regimen is cisplatin + gemcitabine, generally administered in 21-day cycles. Although a randomised, open-label, Phase 3 trial of 1L cisplatin + gemcitabine vs MVAC did not show superior efficacy (HR for OS, 1.09 [95% CI, 0.88–1.34]; HR for PFS, 1.09 [95% CI, 0.89–1.34]), cisplatin + gemcitabine had a more tolerable safety profile [38, 39] and has superseded MVAC in clinical practice in the UK and many other countries.

For patients who are unsuitable for cisplatin but nonetheless suitable for platinum-based chemotherapy, carboplatin + gemcitabine in 21-day cycles is the standard of care and recommended by NICE (Fig. 1) [13, 14]. A randomised Phase 2/3 trial (EORTC 30986) compared carboplatin + gemcitabine with methotrexate, carboplatin, and vinblastine (M-CAVI) in cisplatin-ineligible patients (defined as an ECOG PS of 2 and/or impaired renal function [GFR of >30 but <60 mL/min]). Neither arm was superior in terms of efficacy (HR for OS, 0.94 [95% CI, 0.72–1.22]; *P* = 0.64; HR for PFS, 1.04 [95% CI, 0.80–1.35]), but M-CAVI was more toxic than carboplatin + gemcitabine, particularly in those with impaired renal function [40]. Cisplatin-based chemotherapy is generally considered to have improved efficacy vs carboplatin-based chemotherapy; however, recent studies have shown that differences in efficacy may be less than previously thought and may not be significant [41–43]. For patients with mild renal dysfunction (GFR of 40–59 mL/min), a split dose of cisplatin may be considered (such as 35 mg/m^2^ on days 1 and 2 or days 1 and 8), although the level of evidence is low [4, 44, 45]. Recently, a substudy of the CheckMate-901 Phase 3 trial that enrolled cisplatin-eligible patients showed that treatment with nivolumab + cisplatin-based chemotherapy followed by nivolumab monotherapy resulted in significantly longer OS and PFS than cisplatin-based chemotherapy alone (HR for OS, 0.78 [95% CI, 0.63–0.96]; *P* = 0.02; HR for PFS, 0.72 [95% CI, 0.59–0.88]; *P* = 0.001) [46]. The implications of these results for clinical practice in the UK and other countries are unclear.

Pembrolizumab (anti–PD-1) and atezolizumab have been approved by the European Medicines Agency for patients with advanced UC with PD-L1–positive tumours who are ineligible for cisplatin (Fig. 1) [8, 13]. However, subsequent results from randomised trials have cast doubt on the relative efficacy of 1L ICI monotherapy treatment [10, 11]. As a result, 1L ICI treatment may be considered for patients who are unsuitable for or unwilling to receive platinum-based chemotherapy and who have a PD-L1–positive tumour [8, 13]. Atezolizumab (but not pembrolizumab) is recommended by NICE for 1L therapy in patients with PD-L1–positive tumours (≥5% expression on tumour-infiltrating immune cells; Ventana SP142 assay; Table 1) [47]. This approval was based on data from cohort 1 of the single-arm, Phase 2 IMvigor210 trial of atezolizumab monotherapy in previously untreated, cisplatin-ineligible patients (defined as ≥1 of the following: GFR of >30 but <60 mL/min [Cockcroft-Gault formula], grade ≥2 hearing loss or peripheral neuropathy, or ECOG PS of 2). After a median follow-up of 17.2 months, median OS was 15.9 months (95% CI, 10.4 to not estimable) in all patients and 12.3 months (95% CI, 6.0 to not estimable) in patients with PD-L1–positive tumours [48]. A single-arm, Phase 2 trial (KEYNOTE-052) investigated pembrolizumab in cisplatin-ineligible patients and reported a median OS of 11.3 months (95% CI, 9.7–13.1) [49]; however, pembrolizumab was not recommended by NICE or accepted for use by the SMC in this population based on their assessment of its cost-effectiveness [50, 51]. In subsequent randomised Phase 3 trials (IMvigor130 and KEYNOTE-361), atezolizumab or pembrolizumab administered as monotherapy or in combination with platinum-based chemotherapy did not significantly improve OS vs chemotherapy alone [10, 11], which led to the voluntary withdrawal of the US Food and Drug Administration approvals of pembrolizumab and atezolizumab monotherapy for cisplatin-ineligible patients with PD-L1–positive tumours by the drug manufacturers [52, 53]. Nonetheless, exploratory analyses from the IMvigor130 trial in cisplatin-ineligible patients with high PD-L1 tumour expression suggest that these patients could potentially experience clinical benefit with atezolizumab monotherapy vs chemotherapy (HR for OS, 0.56 [95% CI, 0.34–0.91]) [54]. In the US, the indication for pembrolizumab (but not atezolizumab) was revised to patients with advanced UC who are not eligible for any platinum-based chemotherapy, irrespective of PD-L1 status [53]; however, no prospective study reported to date has evaluated 1L ICI treatment specifically in a platinum-ineligible population. Patients unsuitable for platinum-based chemotherapy may include those with a GFR of <30 mL/min, ECOG PS of >2, ECOG PS of 2 and GFR of <60 mL/min, or grade >2 comorbidities [13], although working definitions for platinum eligibility used in clinical practice may vary.

In the US, the combination of enfortumab vedotin (EV; an ADC targeted to the cell surface protein nectin-4) + pembrolizumab received accelerated approval as 1L treatment for cisplatin-ineligible patients based on results reported in Phase 1b/2 cohorts, including high objective response rates (64.5–73.3%) and median OS (22.3–26.1 months) [55–57]. Recently, it was reported that in the EV-302 Phase 3 trial, EV + pembrolizumab resulted in substantial improvements in PFS and OS vs platinum-based chemotherapy in an all-comer population (HR for PFS, 0.45 [95% CI, 0.38–0.54]; *P* < 0.00001; HR for OS, 0.47 [95% CI, 0.38–0.58]; *P* < 0.00001) [58]. EV + pembrolizumab has not yet received marketing authorisation in Europe. Cost-effectiveness assessments for this regimen will determine its relevance to UK clinical practice.

## 1L maintenance treatment following platinum-based chemotherapy

Avelumab (anti–PD-L1) 1L maintenance is standard of care for patients who have had an objective response (complete or partial) or stable disease after completing 1L platinum-based chemotherapy [8, 13] and is recommended by NICE and accepted for use by the SMC (Fig. 1) [59, 60]. This recommendation is based on results from the randomised, open-label, Phase 3 JAVELIN Bladder 100 trial that compared avelumab 1L maintenance + best supportive care (BSC) vs BSC alone in patients who had received 4–6 cycles of platinum-based chemotherapy (cisplatin or carboplatin + gemcitabine) without disease progression [12, 61]. Patients were enrolled after an interval of 4–10 weeks since their last dose of chemotherapy. Avelumab 1L maintenance was continued until disease progression, unacceptable toxicity, or any other criterion for discontinuation was met. OS and PFS were significantly improved with avelumab 1L maintenance + BSC vs BSC alone [12, 61]. After ≥2 years of follow-up in the overall population, the HR for OS (measured from start of maintenance) was 0.76 (95% CI, 0.63–0.91; *P* = 0.0036), and the HR for PFS was 0.54 (95% CI, 0.46–0.64; *P* < 0.0001) [61]. HRs for OS and PFS were similar in patients who had received cisplatin + gemcitabine or carboplatin + gemcitabine as 1L chemotherapy prior to maintenance, and in patients who had a complete response, partial response, or stable disease with 1L chemotherapy, all favouring avelumab [61]. The observed safety profile of avelumab 1L maintenance was consistent with those seen in previous trials of avelumab [12, 61].

The NICE recommendation specifies that avelumab maintenance should be administered until disease progression or stopped after 5 years of uninterrupted treatment, which is based on economic modelling and the assumption that few patients would remain on treatment at 5 years [60]. However, the SMC does not impose this limit [59, 60]. There are no data defining the optimal duration of treatment with avelumab in this setting. PD-L1 testing is not required to determine eligibility of avelumab 1L maintenance. ESMO guidelines state that carboplatin-based chemotherapy followed by avelumab maintenance is preferred vs 1L ICIs in cisplatin-ineligible patients [8]. Considering the OS benefit seen with avelumab 1L maintenance in the JAVELIN Bladder 100 Phase 3 trial, and the failure to demonstrate superiority with 1L ICIs compared with 1L platinum-based chemotherapy in other Phase 3 trials, this is a reasonable approach for patients willing to undergo chemotherapy and for whom extended survival is a key therapeutic goal.

## Later lines of treatment for advanced UC

Options for subsequent treatment are generally dependent on prior treatment received (Fig. 1). In patients who have received 1L platinum-based chemotherapy and avelumab 1L maintenance, rechallenge with platinum-based chemotherapy may be an option if disease progression occurred ≥12 months after completion of a prior platinum regimen [8, 13]; however, there is no evidence to support this approach, and available alternatives with proven OS benefit may be considered first, where available.

ICIs are approved for patients who have had disease progression during or after platinum-based chemotherapy but remain ICI naive. Recently updated ESMO and European Association of Urology (EAU) guidelines recommend second-line (2L) ICI treatment in patients who have disease progression with 1L chemotherapy and therefore are not eligible to receive avelumab 1L maintenance [8, 13]. In the randomised, open-label, Phase 3 KEYNOTE-045 trial, pembrolizumab was compared with chemotherapy (investigator’s choice of paclitaxel, vinflunine, or docetaxel) in patients with advanced UC that had progressed with platinum-based chemotherapy (any PD-L1 status). The trial met its primary endpoint by showing longer OS with pembrolizumab vs chemotherapy (HR, 0.70 [95% CI, 0.57–0.85]; *P* < 0.001), and the safety profile of pembrolizumab was also more favourable than that of chemotherapy [62]. Pembrolizumab is recommended by ESMO guidelines (level 1 evidence) and accepted for use by the SMC in patients who have received prior platinum-based chemotherapy, with a 2-year stopping rule; however, pembrolizumab is not recommended by NICE based on their assessment of its cost-effectiveness [8, 63, 64]. Atezolizumab is recommended in ESMO guidelines (level 2 evidence) and by NICE for 2L treatment of patients with advanced UC, irrespective of tumour PD-L1 status [8, 65]. This recommendation is based on data from the randomised, open-label, Phase 3 IMvigor211 trial that compared atezolizumab vs chemotherapy (investigator’s choice of paclitaxel, vinflunine, or docetaxel) in patients with advanced UC that had progressed with platinum-based chemotherapy (any PD-L1 status) [66], in addition to a prior single-arm study (IMvigor210 cohort 2) [67]. In the prespecified primary analysis population of patients with PD-L1–positive tumours (≥5% expression on tumour-infiltrating immune cells; Ventana SP142 assay) in the Phase 3 trial, OS was not significantly longer with atezolizumab vs chemotherapy (HR, 0.87 [95% CI, 0.63–1.21]; *P* = 0.41); however, in an exploratory analysis in the overall population (intention-to-treat population with any PD-L1 status), a numerical improvement in OS was observed (HR, 0.85 [95% Cl, 0.73–0.99]) [66]. The safety profile of atezolizumab was favourable compared with that of chemotherapy. NICE guidance states that atezolizumab can be administered for up to 2 years or until disease progression [65].

The randomised, open-label, Phase 3 EV-301 trial evaluated EV vs investigator’s choice of chemotherapy in patients with advanced UC who had received prior treatment with platinum-based chemotherapy and an ICI [68]. In the interim analysis, OS was longer in the EV arm than in the chemotherapy arm (HR, 0.70 [95% CI, 0.56–0.89]; *P* = 0.001). The overall incidence of treatment-related adverse events of any grade and of grade ≥3 was similar in both arms. Based on the results of the EV-301 trial, EV has been approved by the European Medicines Agency for the treatment of patients who have previously received platinum-based chemotherapy and an ICI (PD-1 or PD-L1 inhibitor) [8, 13]. EV has not been recommended by NICE and is not accepted for use by the SMC because relevant data have not been submitted for appraisal by the manufacturer [69, 70]; however, based on available clinical trial data, EV should be considered a standard of care after chemotherapy and ICI treatment, if available.

In Europe, other options for later-line treatment of patients with advanced UC are limited, and evidence of benefit with these options is based on trials conducted prior to the emergence of ICIs. There is a lack of trial evidence to show an OS benefit with 2L or later chemotherapy [71]. Although vinflunine is approved in Europe for the treatment of patients who have received platinum-based chemotherapy [8, 13], it is not recommended by NICE or accepted for use by the SMC [72, 73]. In UK clinical practice, some patients receive 2L weekly paclitaxel monotherapy based on modest efficacy reported in Phase 2 trials [74, 75].

Recently, cohort 1 of the randomised Phase 3 THOR trial showed significantly improved OS and PFS with erdafitinib, a small-molecule FGFR inhibitor, vs chemotherapy (docetaxel or vinflunine) in patients with *FGFR2/3* molecular alterations who had prior ICI treatment (HR for OS, 0.64 [95% CI, 0.47–0.88] *P* = 0.005; HR for PFS, 0.58 [95% CI, 0.44–0.78] *P* = 0.0002) [76]. In contrast, cohort 2 of the THOR trial showed no statistically significant difference in OS between erdafitinib vs pembrolizumab in pretreated patients with *FGFR2/3* molecular alterations without prior ICI treatment (HR, 1.18 [95% CI, 0.92–1.51]) [77]. Erdafitinib is yet to receive marketing authorisation in Europe.

## Areas of uncertainty regarding systemic anticancer treatment for UC

Several questions remain regarding systemic anticancer treatment of UC and treatment sequencing. To date, no prospective trial has assessed the impact on subsequent treatment of the timing of relapse after neoadjuvant or adjuvant treatment. In clinical practice, a reasonable approach (in the absence of clinical data) is for patients who have had disease relapse ≥12 months after the end of neoadjuvant or adjuvant treatment to be treated via the same approach as patients diagnosed with de novo metastatic UC; patients who experience relapse within 12 months should be treated with therapies used in or reserved for the 2L setting. This approach has been used as the basis for inclusion criteria for recent trials in the 1L setting [12, 48, 78]. However, there is currently no evidence that rechallenge with platinum-based chemotherapy has more favourable efficacy than ICI treatment.

For 1L treatment with platinum-based chemotherapy, the optimal number of cycles has not been prospectively studied; however, 4–6 cycles are considered the standard of care based on pivotal trials [8, 13, 79]. A retrospective study found no difference in OS between patients with metastatic UC who had received 3–5 cycles (median, 4) or 6–9 cycles (median, 6) of chemotherapy (HR, 1.02 [95% CI, 0.78–1.33]) [80]. An ongoing, randomised, Phase 2 trial being conducted in the UK, Spain, and France (DISCUS; EudraCT: 2021-001975-17) is evaluating the effect of 3 vs the standard 6 cycles of platinum-based chemotherapy prior to avelumab maintenance on patient-reported outcomes (primary endpoint); the trial will also evaluate efficacy and safety.

The role of PD-L1 testing remains an area of debate in the UC treatment landscape. As discussed, adjuvant nivolumab and 1L atezolizumab in cisplatin-ineligible patients have been approved in Europe only in patients with PD-L1–positive tumours (in different disease stages) [30, 31, 47]. However, in general, PD-L1 expression has been associated with inconsistent predictive value in trials of ICIs. Several trials of ICIs in UC have found some evidence for enrichment of improved outcomes in patients with PD-L1–positive tumours, although efficacy benefits have not been limited to these subgroups [10, 67]. In the KEYNOTE-045 trial, OS analyses favoured 2L pembrolizumab vs chemotherapy in patients with PD-L1–positive or PD-L1–negative tumours [81]. Furthermore, PD-L1 expression in tumours is heterogeneous [82], and assays used to determine PD-L1 status, the cell types assessed (tumour cells and/or immune cells), and cutoffs used to define PD-L1 positivity have varied between trials. Thus, in the absence of validated data to support the use of PD-L1 as a predictive biomarker, it is the authors’ opinion that PD-L1 testing has little demonstrable value in treatment selection for patients with advanced UC beyond practical requirements related to treatment access (Table 1).

Globally, ≈5-10% of patients with UC have tumours that originate in the upper urinary tract (eg, renal pelvis or ureter) [4, 8, 9], although higher incidences are reported in some geographic regions [83]. Upper tract tumours may have different characteristics than lower tract tumours, including a higher incidence of primary tumours being invasive at diagnosis (≈60% vs 15–25% of bladder tumours) [83, 84], which is associated with a worse prognosis. Consequently, the proportion of patients with UTUC is higher in populations with advanced UC than in populations with earlier stages of UC [83, 84]. Next-generation sequencing studies have found differences in the prevalence of several gene mutations in upper tract vs lower tract tumours [84]. Few Phase 3 studies have enrolled only patients with UTUC; therefore, clinical decision-making is generally extrapolated from subgroup analyses of studies in broader UC populations and single-centre studies in UTUC populations. In patients with advanced UC, a retrospective analysis of three randomised trials of platinum-based chemotherapy found that primary tumour location had no impact on OS or PFS [85]. In general, subgroup analyses from trials of ICIs in advanced UC have reported similar efficacy in patients with upper tract vs lower tract tumours [48, 49, 62, 86]. Given the low incidence of UTUC compared with lower tract UC, a global effort is needed to design the next generation of clinical trials in this patient population.

Although most bladder tumours are classified as UC, ≈10% of bladder tumours have non-UC histology, including squamous, small cell, sarcoma, and adenocarcinoma histologies [4]. These rare histologies have generally been excluded from trials that define current practice in UC. Data for systemic anticancer therapy in non-UC bladder cancer are limited; therefore, published guidance recommends that systemic treatment can be based on regimens known to be effective in tumours with a similar histology found in other sites [4]. However, regimens effective for UC often have limited efficacy in patients with non-UC bladder cancers. Further studies are needed to assess effective treatments in these patient populations.

## Future perspectives

In the UK, only 30% of patients with advanced UC receive 1L treatment [87]; across global real-world studies, ≈40% of patients receive 1L treatment, with only 15–20% receiving 2L or later treatment [88–91]. Therefore, a significant unmet need remains, particularly in the 1L setting.

Two novel agents have been approved for later-line treatment of advanced UC outside Europe based on single-arm studies. Firstly, sacituzumab govitecan (ADC targeted to trophoblast cell-surface antigen 2), which has shown activity in patients with advanced UC following disease progression with platinum-based chemotherapy and ICI therapy [92]; an ongoing Phase 3 trial is assessing sacituzumab govitecan vs chemotherapy. Secondly, erdafitinib has shown significantly improved efficacy vs chemotherapy in the randomised Phase 3 THOR trial in a cohort of patients with prior treatment including an ICI [76]. Other ongoing Phase 3 trials of systemic anticancer therapy that are registered on ClinicalTrials.gov are summarised in Table 2. A Phase 3 trial that assessed nivolumab + ipilimumab (anti–cytotoxic T lymphocyte antigen-4) vs platinum-based chemotherapy as 1L treatment for patients with advanced UC did not meet its primary endpoint of prolonged OS in patients with PD-L1–positive tumours [93]. However, as discussed previously, in a substudy from this trial, significantly improved OS and PFS were reported with 1L nivolumab + cisplatin-based chemotherapy followed by nivolumab monotherapy vs cisplatin-based chemotherapy [46]. Considerable improvements in OS and PFS were seen with EV + pembrolizumab vs platinum-based chemotherapy in the Phase 3 EV-302 trial, which enrolled platinum-eligible patients [58]. Results from these trials have the potential to provide additional options in the 1L setting, but the relevance of these regimens to UK clinical practice will depend on regulatory and economic assessments.

**Table 2:** Ongoing Phase 3 trials in UC registered on ClinicalTrials.gov.

Trial name (identifier) [location]	Investigational agent(s)	Treatment arms	Patient population	Primary endpoint
Neoadjuvant treatment				
MK-3475-B15/KEYNOTE-B15/ EV-304 (NCT04700124) [global]	Pembrolizumab + enfortumab vedotin	1. Pembrolizumab + enfortumab vedotin + surgery 2. Cisplatin + gemcitabine + surgery	Cisplatin eligible and eligible for cystectomy + pelvic lymph node dissection	Event-free survival
MK-3475-866/KEYNOTE-866 (NCT03924856) [global]	Pembrolizumab	1. Pembrolizumab + cisplatin + gemcitabine + surgery 2. Placebo + cisplatin + gemcitabine + surgery	Cisplatin eligible	Event-free survival
MK-3475-992/KEYNOTE-992 (NCT04241185) [global]	Pembrolizumab	1. Pembrolizumab + chemotherapy + radiotherapy 2. Placebo + chemotherapy + radiotherapy	Eligible for chemoradiotherapy	Bladder intact event-free survival
CA017-078 (NCT03661320) [global]	Nivolumab	1. Cisplatin + gemcitabine 2. Cisplatin + gemcitabine + nivolumab	Cisplatin eligible	pCR rate and event-free survival
CA045-009 (NCT04209114) [global]	Nivolumab + bempegaldesleukin	1. Nivolumab + bempegaldesleukin + surgery → adjuvant nivolumab + bempegaldesleukin 2. Nivolumab + surgery → adjuvant nivolumab 3. Surgery alone (no neoadjuvant or adjuvant therapy)	Cisplatin ineligible	pCR rate and event-free survival
NCI-2018-03264 (NCT03775265) [US]	Atezolizumab	1. Chemoradiotherapy 2. Chemoradiotherapy + atezolizumab	Eligible for neoadjuvant chemotherapy	Bladder intact event-free survival
NIAGARA (NCT03732677) [global]	Durvalumab	1. Durvalumab + cisplatin + gemcitabine → adjuvant durvalumab 2. Cisplatin + gemcitabine	Cisplatin eligible	pCR rate and event-free survival
VOLGA (NCT04960709) [global]	Durvalumab + tremelimumab + enfortumab vedotin	1. Durvalumab + tremelimumab + enfortumab vedotin → adjuvant tremelimumab + durvalumab 2. Durvalumab + enfortumab vedotin → adjuvant durvalumab 3. Cystectomy with or without adjuvant nivolumab	Cisplatin ineligible/refusal of cisplatin	Efficacy (pCR rate, event-free survival) and safety
SunRISe-2 (NCT04658862) [global]	Cetrelimab + TAR-200	1. Cetrelimab + TAR-200 2. Cisplatin + gemcitabine + radiotherapy	Ineligible for cystectomy	Bladder intact event-free survival
Adjuvant treatment				
IMvigor011 (NCT04660344) [global]	Atezolizumab	1. Atezolizumab 2. Placebo	ctDNA positive following cystectomy	Investigator-assessed DFS
AMBASSADOR (NCT03244384) [US]	Pembrolizumab	1. Pembrolizumab 2. Observation	Prior neoadjuvant chemotherapy or cisplatin ineligible	OS and DFS
1L treatment				
NILE (NCT03682068) [global]	Durvalumab + tremelimumab	1. Durvalumab + gemcitabine + cisplatin or carboplatin 2. Durvalumab + tremelimumab + gemcitabine + cisplatin or carboplatin 3. Gemcitabine + cisplatin or carboplatin	Platinum eligible	OS
BGB-A317-310 (NCT03967977) [China]	Tislelizumab	1. Tislelizumab + gemcitabine + cisplatin or carboplatin 2. Placebo + gemcitabine + cisplatin or carboplatin	Platinum eligible	OS
RC48-C016 (NCT05302284) [China]	Disitamab vedotin + toripalimab	1. Disitamab vedotin + toripalimab 2. Gemcitabine + cisplatin or carboplatin	Platinum eligible	PFS and OS
JS001-038-III-UBC (NCT04568304) [China]	Toripalimab	1. Toripalimab + gemcitabine + cisplatin or carboplatin 2. Placebo + gemcitabine + cisplatin or carboplatin	Platinum eligible	PFS
1L maintenance treatment				
MAIN-CAV (NCT05092958) [US and Canada]	Cabozantinib	1. Cabozantinib + avelumab 2. Avelumab	Disease control with 4-6 cycles of platinum-based chemotherapy	OS
2L or later treatment				
TROPiCS-04 (NCT04527991) [global]	Sacituzumab govitecan	1. Sacituzumab govitecan 2. Paclitaxel, docetaxel, or vinflunine	Progression after prior immune checkpoint inhibitor (including maintenance) and platinum-based chemotherapy	OS
NCI-2020-07651/S1937 (NCT04579224) [US]	Eribulin	1. Eribulin 2. Eribulin + gemcitabine 3. Paclitaxel, docetaxel, or gemcitabine	Progression after prior immune checkpoint inhibitor, platinum-based chemotherapy, and enfortumab vedotin	OS

*1L* first line, *2L* second line, *ctDNA* circulating tumour DNA, *DFS* disease-free survival, *OS* overall survival, *pCR* pathological complete response, *PD-L1* programmed death ligand 1, *PFS* progression-free survival, *UC* urothelial carcinoma.

## Conclusions

The information summarised in this review provides an overview, from a UK perspective, of key data and clinical developments that support the current standard of care for systemic treatment in patients with UC. Platinum-based chemotherapy remains the cornerstone of systemic treatment for patients with UC. However, the treatment landscape has and continues to evolve rapidly with the development of several new treatments, including different ICIs and ADCs, which have been shown to provide long-term clinical benefits in different populations. Although treatment options available in the UK and other countries depend on local approvals and reimbursement decisions, these developments have improved the prognosis for patients with UC. Despite this, outcomes remain poor overall, particularly in patients with advanced UC; therefore, it is imperative that eligible patients receive optimal treatment at each decision point.
